# The Catastrophic Effects of Psychogenic Polydipsia: A Case Report

**DOI:** 10.7759/cureus.44766

**Published:** 2023-09-06

**Authors:** Davin J Evanson, Eduardo D Espiridion

**Affiliations:** 1 Division of Diagnostic Radiology, Drexel University College of Medicine, Wyomissing, USA; 2 Psychiatry, West Virginia School of Osteopathic Medicine, Lewisburg, USA; 3 Psychiatry, Drexel University College of Medicine, Philadelphia, USA; 4 Psychiatry, Philadelphia College of Osteopathic Medicine, Philadelphia, USA; 5 Psychiatry, Reading Hospital - Tower Health, West Reading, USA

**Keywords:** water intoxication, delirium, schizophrenia, hyponatremia, psychogenic polydipsia

## Abstract

Patients with hyponatremia are at risk of severe complications including seizures, coma, and death. Psychiatric patients are particularly susceptible to death from hyponatremia due to the association between psychiatric conditions and psychogenic polydipsia, characterized by water intoxication. We report a case of a schizophrenic patient who presented with altered mental status, leading to a differential diagnosis narrowed through clinical investigations to include hypovolemic hyponatremia, syndrome of inappropriate antidiuretic hormone secretion (SIADH), and psychogenic polydipsia. This case underscores the need to inquire about schizophrenic patients' water intake, advocating for a standardized approach. The timely diagnosis of disorders causing electrolyte abnormalities can prevent severe complications and aid in the management of psychiatric patients.

## Introduction

Polydipsia increases the risk of death by 74% in patients with schizophrenia [1]. Polydipsia is characterized by excessive thirst and fluid consumption, leading to polyuria with diluted urine [2]. An association between polydipsia and psychiatric disorders, especially schizophrenia, has been well-established. Recent studies indicate that between 11-20% of patients with schizophrenia have associated psychogenic polydipsia [3,4]. Among patients with this association, approximately 20% will develop severe hyponatremia, a potentially fatal condition [3]. Severe hyponatremia, presenting a risk of seizures, occurs in about 5% of cases, as demonstrated in the case discussed in this report [5].

## Case presentation

A 71-year-old male with a past psychiatric medical history that included schizoaffective disorder-bipolar type and anxiety was brought to the emergency department (ED) of a community hospital by emergency medical services (EMS) due to altered mental status. Prior to the arrival, he was described as having abnormal and agitated behavior and had been unable to answer questions appropriately. He was described as having visual hallucinations. When the EMS found him, he had been unconscious with hematemesis on his chest. Upon arrival, the patient was aphasic and unresponsive to commands. The symptoms were concerning initially for stroke. Stroke precautions and appropriate medical care, including the administration of 4 grams of magnesium sulfate in the ED, were provided. Both CT and MRI returned normal results. Therefore, an acute stroke was deemed less likely, leading to concerns for unspecified delirium. The key laboratory findings are presented in Table 1.

**Table 1:** Relevant laboratory findings

Laboratory test	Results	Reference values
Serum Na+	115 mmol/L	136 – 145 mmol/L
Serum K+	4.0 mmol/L	3.5 – 5.1 mmol/L
Serum Cl-	86 mmol/L	98 – 107 mmol/L
Serum creatinine	0.78 mg/dL	0.60 – 1.30 mg/dL
Serum Mg2+	1.3 mg/dL	1.9 – 2.7 mg/dL
Lactic acid	1.8 mmol/L	0.6 – 1.4 mmol/L
Serum osmolality	246 mOSM/K	280 – 290 mOSM/K
Urine osmolality	280 mOSM/K	300 – 1200 mOSM/K
Urine Na+	16 mmol/L	>20 mmol/L
Urine urea	347 mg/dL	310 – 3300 mg/dL
Urine creatinine	51.9 mg/dL	20 – 320 mg/dL
Lithium	0.1 mmol/L	0.6 – 1.2 mmol/L
Thyroid function test	0.644 ulU/mL	0.450 – 5.330 ulU/mL
Glucose	186 mg/dL	70 – 100 mg/dL
Hemoglobin	11.4 g/dL	13.8 – 17.2 g/dL
Platelets	212,000	150,000 – 450,000
White blood cell count	12.1 × 10^9^/L	4.5 – 11.0 × 10^9^/L
Hematocrit	32.1%	41% – 50%

**Figure 1:**
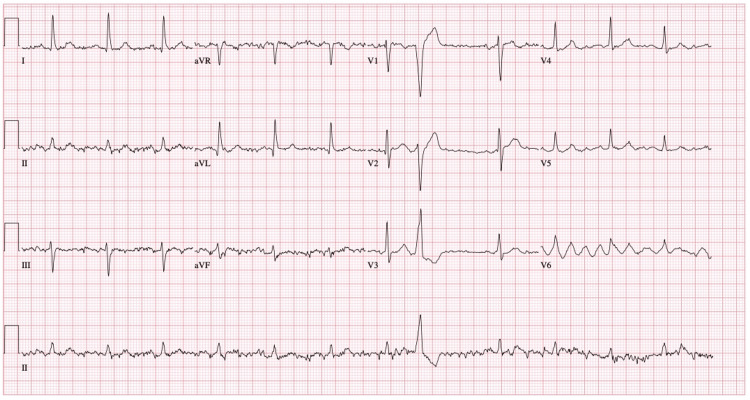
Initial EKG shows normal sinus rhythm with premature ventricular complexes EKG: electrocardiogram

**Figure 2:**
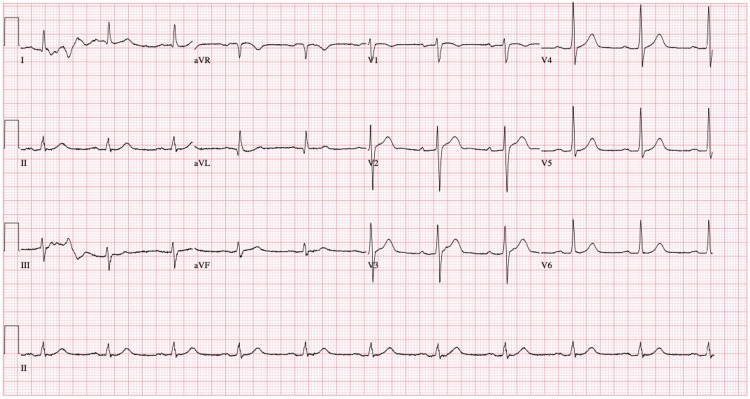
Repeat EKG shows sinus rhythm with first-degree atrioventricular (AV) block EKG: electrocardiogram

Electrocardiogram (EKG) findings revealed sinus rhythm with premature ventricular complexes and a nonspecific ST abnormality (Figure 1). An EKG taken seven hours later showed sinus rhythm with first-degree atrioventricular (AV) block (Figure 2). Subsequently, the patient was admitted to the ICU. Collateral information provided the medical team the details about the patient's medication regimen, which comprised lithium 300 mg three times daily for four years, lorazepam 1 mg nightly, and paliperidone palmitate 234 mg/1.5 mL syringe once monthly. Additionally, the patient had a history of hyponatremia attributed to excessive fluid intake, with a reported consumption of 16-17 glasses of water per day.

The diagnoses that were primarily considered were hypovolemic hyponatremia, syndrome of inappropriate antidiuretic hormone secretion (SIADH) based on the patient’s initiatory laboratory workup. Psychogenic polydipsia was also considered a plausible diagnosis, given the patient's psychiatric history. To differentiate between each, certain criteria were considered by the medical team. SIADH can be caused by certain medications and presents with euvolemic hyponatremia, low serum osmolality, high urine osmolality, and low urine output with concentrated urine. The patient had a low urine osmolality but met all other criteria including the use of lithium. Hypovolemic hyponatremia presents with low urine sodium, high urine osmolality, low or normal serum osmolality, and clinical signs of dehydration, which can be caused by emesis. Psychogenic polydipsia presents with diluted urine with low urine osmolality in conjunction with excessive fluid intake, a psychiatric diagnosis, and an abnormally high urine output. The patient had an unknown urine output and a collateral history of excessive fluid intake. Therefore, a diagnosis based on the initial workup was difficult due to overlapping presentations and the patient not meeting the complete criteria for any particular diagnosis.

The patient's hospitalization was further complicated by the appearance of a tremor approximately eight hours after presentation, accompanied by 4700 mL of urine output. Intubation was necessary for airway protection and due to concerns regarding aspiration pneumonia owing to active emesis and obtundation. An electroencephalogram (EEG) revealed generalized background slowing indicative of diffuse cerebral dysfunction, possibly due to toxic, metabolic, and multifocal brain abnormalities. Encephalopathy emerged as the primary concern. The EEG indicated no seizure activity, attributing tremor to hyponatremia with the potential for resolution following electrolyte correction. The patient received 0.9% normal saline at a rate of 100 mL/h, resulting in a sodium increase to 125 mEq/L within 12 hours. Desmopressin acetate (DDAVP) was administered to slow the correction rate. To prevent overcorrection, isotonic intravenous fluid (IVF) hydration was halted, and D5W was introduced after correction was achieved.

After 16 hours of intubation, the patient self-extubated and expressed a need for water. Upon becoming alert and oriented, the patient provided some history, acknowledging excessive pre-admission fluid consumption due to thirst and dry mouth. Fluid restriction was initiated while allowing for self-correction. Two days later, urinary retention raised concerns for post-obstructive diuresis, leading to the placement of a Foley catheter that yielded a 6000 mL output in five hours. The treatment involved avoiding all IV fluids and limiting fluid intake to 2 L. Additional collateral sources supported the patient's history of medication non-adherence, prolonged counseling center attendance, and a baseline characterized by delirium interspersed with periods of somnolence and lucidity.

Ultimately, the patient was diagnosed with psychogenic polydipsia due to polyuria as the causative factor for hyponatremia. The diagnosis of psychogenic polydipsia was established through a multi-specialty approach, supported by clinical evidence. The patient's excessive urine output (polyuria), accompanied by a strong obsession with fluids and excessive intake, provided the critical indicators. A positive response to fluid restriction emphasized a psychological origin for polyuria, reinforced by the patient's history of psychiatric conditions and the observed periods of delirium. Together, these clinical signs, along with the laboratory results and symptomology, solidified the diagnosis of psychogenic polydipsia as the most fitting explanation for the patient’s hyponatremia.

## Discussion

The diagnosis of psychogenic polydipsia can be challenging due to potential overlap with other conditions with similar clinical features [6]. However, prompt recognition is crucial to avoid potentially life-threatening complications [7]. Similar cases, as observed in other reports, have depicted events resembling the case under discussion, including seizures and aspiration pneumonia [8,9]. Studies have also documented outcomes such as crural compartment syndrome, rhabdomyolysis, and even death [10-12]. In this specific case, a seemingly uncomplicated presentation of hyponatremia turned into a complex diagnostic challenge, with the underlying cause ultimately attributed to psychogenic polydipsia. Of note, while the patient acknowledged excessive fluid intake, he did not perceive it as inappropriate, highlighting the intricate nature of this disorder.

Management of these cases of psychogenic polydipsia is complicated by the intricate nature of care required for psychiatric patients, especially considering medication-induced side effects like SIADH. In our case, the patient was on lithium, a medication with diverse effects on fluid balance and electrolyte regulation. Lithium is associated with adverse effects like polyuria and polydipsia in up to 70% of patients [13]. Although usually linked with SIADH, the relatively low lithium level in our patient introduced an element of complexity, rendering SIADH to remain a diagnostic consideration. The presence of urinary retention further complicates the situation by increasing the risk of water intoxication due to the inability to eliminate excess water [14].

The treatment approach toward psychogenic polydipsia has exhibited significant variability over time, involving a range of interventions. Medication-based strategies have yielded inconsistent results, with recent evidence suggesting the potential efficacy of substances like clozapine and vasopressin type 2 antagonists [15,16]. Non-pharmacological options, such as behavioral modifications, have also been explored as potential therapeutic avenues for managing this complex condition [17].

Notably, our patient had previously admitted to excessive fluid intake and had experienced hyponatremia in the past. As highlighted in other reports, water intake should be a topic of standard inquiry when encountering schizophrenic patients [18]. In our case, the patient had a history of excessive fluid intake. which could have led to earlier clinical suspicion of psychogenic polydipsia had this information been accessible. Due to the relatively high co-occurrence of schizophrenia and psychogenic polydipsia, a simple standard inquiry could help identify up to one out of every five patients at risk of hyponatremia and potentially prevent the catastrophic effects that occurred in this patient.

## Conclusions

The complex interaction between psychiatric disorders, medication regimens, and fluid-electrolyte imbalances can give rise to potentially life-threatening consequences. Thorough history-taking, collateral information, and meticulous clinical investigations are crucial for accurate diagnosis and appropriate interventions. As this case demonstrates, a multifaceted approach involving early clinical suspicion, medication adjustment, and fluid restriction can help effectively manage psychogenic polydipsia and prevent its catastrophic consequences.
